# Implementing COVID-19 Surveillance Through Inter-Organizational Coordination: A Qualitative Study of Three Cities in Colombia

**DOI:** 10.1093/heapol/czab145

**Published:** 2021-12-07

**Authors:** Simon Turner, Carolina Segura, Natalia Niño

**Affiliations:** School of Management, University of los Andes, Bogotá, Colombia; School of Management, University of los Andes, Bogotá, Colombia; School of Medicine, University of los Andes, Bogotá, Colombia

**Keywords:** care coordination, care integration, inter-organizational, coordination, public health, COVID-19, qualitative research

## Abstract

Introducing comprehensive surveillance is recommended as an urgent public health measure to control and mitigate the spread of COVID-19 worldwide. However, its implementation has proven challenging as it requires inter-organizational coordination among multiple health care stakeholders. The purpose of this study was to examine the role of soft and hard mechanisms in the implementation of inter-organizational coordination strategies for COVID-19 surveillance within Colombia, drawing on evidence from the cities of Bogotá, Cali and Cartagena. The study used a case study approach to understand the perspectives of local and national authorities, insurance companies and health providers in the implementation of inter-organizational coordination strategies for COVID-19 surveillance. 81 semi-structured interviews were conducted between June and November 2020. The data was analysed by codes and categorized using New NVivo software. The study identified inter-organizational coordination strategies that were implemented to provide COVID-19 surveillance in the three cities. Both soft (e.g. trust and shared purpose) and hard mechanisms (e.g. formal agreements and regulations) acted as mediators for collaboration and helped to address existing structural barriers in the provision of health services. The findings suggest that soft and hard mechanisms contributed to promoting change among health care system stakeholders and improved inter-organizational coordination for disease surveillance. The findings contribute to evidence regarding practices to improve coordinated surveillance of disease, including the roles of new forms of financing and contracting between insurers and public and private health service providers, logistics regarding early diagnosis in infectious disease, and the provision of health services at the community level regardless of insurance affiliation. Our research provides evidence to improve disease surveillance frameworks in fragmented health systems contributing to public health planning and health system improvement.

## Introduction

The widespread impact of the COVID-19 pandemic has made implementation of public health surveillance an urgent strategy of public health authorities to control and mitigate the disease within health systems internationally (Randazzo et al., 2020). Surveillance of COVID-19 aims to enable rapid detection, isolation, testing, and monitoring of trends in cases and deaths; to identify, follow-up and impose quarantine upon potential contacts; to detect and contain clusters and outbreaks; to monitor epidemiologic trends; and to contribute to understanding of the virus in different environments (World Health Organization, 2020). Epidemiological public health surveillance can also generate real-time data for decision-making on disease monitoring and control (Ibrahim, 2020). However, implementation of surveillance strategies in the context of a sanitary emergency has challenged health systems with varying resources internationally (Dalglish, 2020). One hypothesis for explaining such difficulties is that effective coordination among the different organizations within health systems is required to support adaptation to COVID-19 (Legido-Quigley et al., 2020). This paper outlines two propositions concerning the influence of structural (hard) and motivational (soft) mechanisms on coordination between health system organizations and examines their relevance in the coordination of public health surveillance of COVID-19 in Colombia.

## Mechanisms influencing inter-organizational coordination

The emphasis on improving coordination, or how different organizations work together in the joint planning and delivery of care, is not new and has emerged in previous policy work on improving health systems, e.g. the longstanding agenda for coordinating care across institutions (Rummery, 2009, Shigayeva et al., 2010). Increased coordination of care has been subject to considerable research, including suggested barriers and enablers (Cameron et al.,

2014, Vargas et al., 2016), and recommendations aimed at policymakers for supporting coordinated change (Best et al., 2012).

In evaluating the relevance of previous research on improving coordination arrangements to the challenges posed by COVID-19, we suggest that two qualifications are needed. First, health system changes in response to COVID-19 have been undertaken rapidly against a background of emergency or ´crisis´ planning. Much of the health systems change literature has assumed judicious planning of change over a much longer time interval (e.g. assembling a robust evidence base and social coalition for change) (Turner et al. 2016a; Turner et al. 2016b; Best et al. 2012). Little is known about how temporal constraints of introducing change in a context of emergency influences their planning and implementation. Second, the mechanisms of change in an emergency context, particularly the roles of and interplay between ´softer´ and ´harder´ mechanisms of change, could differ from those typically relied upon during ´care as usual´ (Wensing et al., 2020).

Different perspectives on the key factors that influence coordination arrangements between institutions exist. One relates to structural challenges or impediments to coordinating different institutions within health systems. In relation to Latin America, structural difficulties of implementing comprehensive public health measures among different health systems have been suggested (Giovanella et al. 2020). Structural challenges include coordinating collective surveillance programs (e.g. community strategies) with individual interventions (e.g. clinical treatment), prioritising population needs over market rules, and encouraging collaboration between organizations that normally compete for patients (Giovanella et al., 2020).

To address such structural challenges, some of the literature on health system coordination emphasizes “hard” interventions that influence formal relationships within health systems. These include administration and regulatory change that promotes collaboration (Auschra, 2018), formalization of relationships (Casey, 2008), including inter-organizational contracting arrangements, and changes to the financing of organizations, including the provision of financial incentives linked to quality standards (Turner et al. 2016a). In response to the COVID-19 pandemic, emergency planning and preparedness, including the availability of slack resources, is hypothesized to support necessary adaptation of roles and responsibilities across the health system (Boin and Van Eeten 2013).

Proposition 1: Structural barriers, e.g. market-like incentives, encourage health system fragmentation and undermine coordination among organizations

A structural perspective on coordination only considers structural properties of health systems, and potential impediments arising from these. Another perspective on coordination emphasises behavioural or motivational factors, including “soft” incentives such as “shared purpose” triggered in response to the significant threat to life posed by COVID-19. “Soft” mechanisms of change refer to interpersonal or social processes that encourage shared commitment among organizations to a proposal for change. Inter-organizational collaborations can be conceptualised in an interpersonal way as “a negotiation between people from different organizations with a commitment to working together to secure improvements which could not have been achieved by acting alone” (Dickinson, 2010: 815). Softer mechanisms include exercising political skill and leadership (Wildridge et al., 2004, Casey, 2008, Clarke et al. 2021), building trust and faith among collaborators (Huxham, 2003, Zuckerman et al., 1995), mutual successes and task achievement (Vangen and

Huxham, 2003), ambition and authenticity, information exchange, interpersonal communication, a balance of power, sharing resources (Aunger et al., 2020), and stakeholder involvement, notably of powerful actors with the ability to stymie change (Best et al. 2012). In response to COVID-19, the emergency context might enrich interpersonal commitment in representing a ´burning platform´ that requires rapid action to be addressed (Turner et al. 2021). For example, a study of a US academic medical centre found that the pandemic had become a ´catalyst´ for improved teamwork among the centre´s staff (Srinivasan et al., 2020).

Proposition 2: "Soft" motivational incentives, e.g. shared purpose, encourage informal coordination among organizations, even where structural incentives are lacking.

Taking into account these propositions about improving coordination arrangements between organizations, this paper addresses the following research question: what were the relative roles of structural and motivational incentives in shaping coordination arrangements among organizations to enable COVID-19 surveillance? It addresses this question using interviews with health system stakeholders in Colombia concerning how they perceived, and engaged in, inter-organizational collaboration to support surveillance of COVID-19.

**The health system in Colombia**

This study of the implementation of COVID-19 surveillance strategies is contextualized in the Colombian health system. Table 1 describes characteristics of the Colombian health system and its response to COVID-19. It is composed of actors at multiple levels: public health surveillance is provided by departments and municipalities; healthcare provision is the responsibility of private and public insurers; national surveillance is the responsibility of the national institute of health; and health regulations and insurance contracts are enforced by the national Ministry of Health. Due to the responsibilities of national bodies, there are similarities across local contexts due to their compliance with national surveillance and regulation; however, potential differences arise locally in public health surveillance and health service provision depending on the actions of local government and insurance companies.

INSERT TABLE 1 HERE

The Colombian health system has been characterized as fragmented, segmented and market-oriented (Hernández, 2002, Jaime, 2016). It has faced multiple dilemmas in recent years including financing arrangements, appropriate access to health care, medical autonomy, and balancing the mix of public and private providers (Alvarez-Rosete and Hawkins, 2018 Amaya-Lara et al. 2016; Atun et al. 2015; Garcia-Subirats et al. 2014; Vargas Bustamante and Méndez, 2014). However, recent policy developments prior to COVID-19´s arrival promoted the strengthening of leadership of coordination arrangements between institutions, including "integrated health care networks" (Vargas et al. 2018; Vargas et al. 2015; Vázquez et al. 2015). From a structural perspective, the fragmentation of the system, both in service provision and in their planning, has been theorized to limit the speed of implementation of widescale epidemiological surveillance systems (Giovanella et al. 2020).

**COVID-19 surveillance in Colombia**

At a global level, epidemiological surveillance measures for infectious diseases have focussed on preventive strategies such as quarantine, early detection of cases, and isolation. Surveillance data on positive events have been used for informing decision-making on adjusting public health actions during outbreaks of epidemics worldwide (Declich et al. 1994;

Centers for Disease Control and Prevention 1988). Even though Colombia has long required public health surveillance of infectious diseases such as dengue, malaria, leishmaniasis, chagas, zika and chikungunya, the unprecedented nature, scale and threat to life posed by COVID-19 represented a particular challenge for implementing public health surveillance. Like many countries internationally, Colombia promoted the WHO key COVID-19 recommendations for public health surveillance (World Health Organization, 2020).

The declaration of a national state of emergency in Colombia in March 2020 (Republica de Colombia, 2020a) allowed for greater flexibility over the financing of health services, including additional State funding, and the performance of individual and collective organizational roles within the health system. Drawing on the flexibility provided by the national state of emergency, the Ministry of Health published a national contingency plan to respond to the pandemic in April 2020 (Ministerio de Salud y Protección Social, 2020). Further national resolutions (e.g. Republica de Colombia 2020b) empowered local authorities at city and department level to take actions necessary to preserve public order and guarantee the health of their inhabitants. To support local public health responses to COVID-19, local authorities were encouraged to develop formal agreements among health system stakeholders within their locality to support coordinated actions in response to COVID-19. Formal agreements were reached in the localities studied in April (Cartagena), May (Cali), and September 2020 (Bogotá).

The need to formalize coordination between these actors was based on two factors. Firstly, insurance companies´ responsibilities were limited to their affiliated users; they were not able to test and trace associated cases that had different insurance affiliations. In the majority of cases, a household, cluster or a specific geographical area of risk required the participation of multiple insurance companies to attend and assess members of their particular insurance scheme (Figure 1). Secondly, the national health institute and the local health secretaries were unable to implement timely surveillance without collaboration with insurance companies nationally, which were often the first actors to be informed of potential cases. Even though these two actors were responsible for the surveillance of nationally notifiable diseases, including COVID-19, regardless of insurance affiliation, case numbers exceeded the testing and tracing capacity of these actors early in the pandemic, stimulating awareness of the need for improving inter-organizational collaboration. Comprehensive data on the effectiveness of domiciliary surveillance could not be identified; however, an executive manager of one national insurance provider indicated in a newspaper interview that 82% of positive cases were identified and monitored at home, without the need for hospital attendance (Portafolio, 2021).

INSERT FIGURE 1 HERE

**Materials and Methods**

This paper is derived from a qualitative study of the coordination of responses to COVID-19 within the Colombian health system (Turner and Niño, 2020). We undertook case studies (Yin, 2013), using a combination of descriptive and theory-driven approaches, to understand how stakeholders involved in the planning and delivery of health responded to the pandemic in Colombia and perceived inter-organizational collaboration in such responses (e.g. coordination of surveillance across localities). The Consolidated Criteria for Reporting Qualitative Studies checklist was used for reporting study design, data collection and analysis, and findings (O'Brien et al., 2014).

**Participants**

The findings are based on a dataset of 81 semi-structured interviews conducted between June and November 2020. In total, 114 potential participants were approached for interview; the participation rate was 71 per cent. No participants withdrew from the study once they had participated in an interview. Diverse health system stakeholders at national level and in the three cities were included (Table 2). We included interviews with stakeholders that worked at national governmental bodies directly involved in the planning of COVID-19 response (n=9). In each city, we interviewed representatives of the local government involved in health planning and delivery (n=22), representatives of health insurance companies (n=13), and representatives of hospitals and clinical laboratories (n=37).

INSERT TABLE 2 HERE

**Data collection**

The study participants were recruited using purposive (Palinkas et al. 2015) and snowball sampling (Lewis-Beck et al., 2004). To inform purposive sampling, we mapped key organizations and stakeholders involved in the response to the emergency at the national level and the three cities. All participants contacted had senior positions and were able to provide an overview of their organization's experiences of COVID-19. The stakeholders approached were highly involved in decision making on COVID-19, especially during the period of data collection (the first six months of the emergency in Colombia). Interviewees were asked if they were able to provide contact details for other relevant stakeholders and/or if they could leave us in touch with them. Using snowball sampling (Lewis-Beck et al., 2004) facilitated participants´ willingness to be interviewed. Having mapped relevant stakeholders at the outset of the study helped us to balance potential sampling bias derived from snowball sampling.

Given that data collection took place during the Colombian "lockdown", all the interviews were conducted virtually using online platforms: Microsoft Teams (n=78), Zoom (n=2), and Google Meet (n=1). Microsoft Teams is the platform the university uses the most for online meetings and the one we considered the safest in terms of privacy: 1) it notifies all participants when recording and 2) recordings are only available to the people on the call or people invited to the meeting. Institutional emails were used to create the links for the interviews so participants were protected by the University privacy policy. In the cases of Zoom and Google Meet, the participant chose the platform of their preference; participants considered it was easier for them to connect to the meeting if they used a platform they already knew.

Ethics approval for this research was received from the research ethics committee at the University of los Andes (1166- 2020). All participants provided informed consent before being interviewed and agreed to be recorded. Audio files were saved using a unique code; all transcriptions were anonymised by replacing participants' names with codes. A topic guide informed the interviews (supplement a); however, the questions were adapted to each interviewee's context (Patton, 1990).

**Analysis**

The data was analysed thematically using inductive and deductive approaches (Bradley et al., 2007). All the interviews were transcribed verbatim and coded using New NVivo software. Deductively, the research team coded the interviews following ten main codes and twenty-two sub-codes that were derived from the study objective and its theoretical framework.

Inductively, emerging categories were identified that could help us understand COVID-19 surveillance and inter-organizational coordination in the particular context of the Colombian health system (Table 3).

INSERT TABLE 3 HERE

## Results

The results are organized into two themes. First, we examine perceived structural barriers to the implementation of coordinated work between health secretaries, insurance companies, and health providers. Second, we explain how the pandemic came to represent among participants a stimulus for actors to work together, leading to the development of strategies that relied on inter-organizational coordination to perform COVID-19 surveillance.

### Structural barriers to inter-organizational coordination

Inter-organizational coordination between the health secretaries, insurance companies, and health providers to implement surveillance measures was described by participants as novel: there was a lack of previous collaborative relationships through which these actors shared responsibilities, financial resources and human resources. Structural barriers were identified that had impeded such coordination within the health system historically.

Fragmentation among health care actors

A perceived structural barrier to coordination was fragmentation among different health care actors, both nationally and locally. At the national level, a stakeholder pointed out that negotiating and reaching agreements between the key actors of the health system was often challenging as the result of health system fragmentation. Fragmentation was apparent in the co-existence of different insurance schemes, and different health providers, that could belong to the public or the private sector. Reaching an agreement between insurance companies and local health secretariats was described by one national level participant as a necessity to cope with fragmentation:

> *"We also have to take into account that this situation, that is, that the health system is segmented and fragmented (...) there are rules for the subsidized, the contributive, the military forces, the magisterium. In addition, we deal with the fragmentation of responsibilities, this is something that had an impact in the negotiations" (Ministry of Health representative, SH-A-023).*

Under the national model of "managed competition", health insurers and providers viewed one another as "competitors" that were not obliged to work together:

> *"The [insurance companies] view each other with suspicion, because they are enemies from a commercial point of view; it is a perfectly normal situation in a market that is tight; the same thing happened with hospitals " (Secretary of Health representative, Bogotá, SH-B-025).*

Volume and diversity of provider organizations

The volume and diversity of insurance companies and health providers inhibited their coordination across each city. Providers were diverse in terms of the scale of their infrastructure, their administrative systems, processes and information management. An interviewee from Cali described the difficulties of coordinating seventeen insurance companies operating in the city, stressing the need for a leader that could help these organizations to reach shared agreements:

*"I do believe that there is a need for a health insurances' coordinator at the local or national level, one who heads those organizations and puts them into a regime because the agreement has worked but it is exhausting, that is, there are 17 [insurers], and as I said, they do not have the same performance" (Secretariat of Health representative, Cali, SH-C-008).*

Arm´s length relationships among organizations

Arm´s length relationships between planners and providers of care contributed to distrust between the health secretaries, insurance companies and health providers. The planning secretariats would often play the role of auditing providers´ services including the conditions of their infrastructure, with the consequence that the relationship between planners and providers was confined to narrow bureaucratic and administrative procedures, with the threat of sanctions:

*"The Secretariat is often calling us to close our doors, to assess if we are accomplishing the secretariat guidelines and permits needed to keep the services open. Never to say, ´hey let´s have a conversation´" (Secretariat of Health representative, Bogotá, SH-B-003).*

History of unfulfilled contractual arrangements

Unfulfilled contractual agreements for the delivery of health services also contributed to mistrust between actors. In Cartagena, for example, hospitals feared that costs associated with the treatment of COVID-19 patients would not be reimbursed due to economic debts that had not been paid by local authorities historically, undermining their willingness to take on new responsibilities:

*"It is necessary that they regain out trust, not respect because they will always have respect for representing the State, but trust. And to regain trust, they have to put an end to that indifferent behavior regarding their financial obligations that they acquire with us" (Hospital director, Cartagena, SH-D-002).*

Poorly defined organizational roles and relationships

Organizational roles in response to COVID-19 were poorly defined, initially, due to a lack of communication among the diversity of actors within the health system. This contributed to uncertainty and tensions over which organizations should carry out and finance particular public health functions such as "testing and tracing" during the early stage of the pandemic, as this example from Cali shows:

*"Initially, the national government designated that the health secretariats were going to be in charge of testing, and that it was going to be for free. This was April or May [2020]. Suddenly, the government said it was the [insurance companies'] responsibility. There was not a clear decision from the beginning. It was not clear if it was a public health entity the responsible and all the resources had to come from [a fund for emergency mitigation] – all the resources were coming from the government - or is it was funded by capitation and services delivered like any other disease" (Hospital Director, Cali, SH-C-007).*

In summary, coordination and cooperation among health providers was not a usual practice in Colombia prior to COVID-19´s arrival. The interviewees stated that the relationships between insurers, service providers, both public and private, and local authorities were minimal and restricted to specific activities. They described multiple structural barriers for coordination within the health system. However, the pandemic was seen as a trigger for addressing some of the structural barriers to coordination just described.

**Mechanisms of inter-organizational coordination to improve COVID-19 surveillance**

The coordination of actors to undertake COVID-19 surveillance (as shown in Table 4) was underpinned by hard and soft coordination mechanisms.

INSERT TABLE 4 HERE

Hard mechanism: formal agreements clarified roles, responsibilities, and resource use

In order to implement a timely surveillance strategy for COVID-19, the three cities studied developed formal agreements between the health secretaries, the insurance companies and health providers, including laboratories, for COVID-19 testing. In the case of Bogotá and Cali, the strategy was called "Convenio Tripartita" ("Tripartite Agreement"), while Cartagena introduced a similar strategy called "Cuidemonos Cartagena". The formal inter-organizational agreements defined their respective functions, as well as shared responsibilities and financial resources for responding to COVID-19:

> "*We made the decision to privilege the home care of patients, not only as a logical way to care for these people, but as a way to prevent patients from reaching the emergency services en masse and congesting them. However, to do that home care, we had to make a common pool of resources with the insurers. This is a massive change for us" (Health Secretariat representative, Bogotá, SH-B-025).*

The inter-organizational agreements had three purposes. First, to enable diagnostic testing of all members of a family or community, irrespective of their insurance affiliation. Some interviewees stated that coordination approaches to testing were needed because a single organization did not have the resources or the capacity to carry out these activities. The insurance companies were able to test their clients, but they could not guarantee that other suspected cases in the same cluster covered by other insurance companies could be tested, meaning they could not perform contact tracing or control the local risk of exposure.

Second, the agreements improved the collection and sharing of data for facilitating early diagnosis, performing active case identification, contact tracing and monitoring, and centralised data management and surveillance. Data management for public health surveillance, traditionally implemented by the health secretariats and national health institute, was widened to include other actors, including health providers and insurance companies. In Cali, a "passive" approach to epidemiological surveillance was adopted historically. The pandemic, in stimulating shared data management between the secretariat and the health providers, enabled an "active" approach to the search for cases in specific households and neighbourhoods.

> *"The epidemiological surveillance group as it was historically used routine surveillance. I would say that a passive surveillance where reports were made from the institutions, but not an active surveillance that is the search, which is behind the cases and identify cluster and test suspected cases" (Insurance company representative, National level, SH-C-008).*

Third, the agreements supported coordinated surveillance and educational work with local communities. In Cartagena, for example, a programme called Heroic Neighborhood ("Barrio Heroico", in Spanish) fostered the surveillance at the community level under the guidance of the District's Health Secretariat and an alliance of insurance companies. The programme's features included an educational programme to promote health literacy, compliance with isolation measures, mask use, social distancing, and COVID-19 surveillance measures within Cartagena´s local communities (Alcaldía Distrital de Cartagena, 2020).

Hard mechanism: declaration of national state of emergency

Actors´ willingness to enter such agreements was supported by the declaration of the state of emergency nationally (República de Colombia, 2020a). The state of emergency decree established the regulations that allowed health organizations to act more flexibly and to transform rapidly their service offerings, mobilize resources, and contract with the state to respond to the emergency. The national declaration allowed the actors to negotiate their interests, align existing service delivery activities, and involve each organization's internal legal and administrative procedures to plan and implement the initiatives. Even though participants described the early stage of planning and implementation as both challenging and time consuming, they agreed that once they had settled on a "common purpose" and financing of the agreement, this allowed the different actors to move forwards collectively with improving COVID-19 surveillance:

> *"We endorsed the agreement, and we invested a significant amount of money on it. The District also added resources of their own. Then we had to face some challenges such as making everyone agree, setting an agreement with the District, making the lawyers [of the different organizations] reaching an agreement, setting a common purpose for all the insurance companies (which are the competition), finding the IPS [health providers] providing the services as part of the agreement, and consolidate a*

*system of information. We had several challenges, but slowly we moved forward" (Insurance Company representative, National level, SH-A-007)*

Soft mechanism: inter-organizational communication and interaction

With regard to softer mechanisms of change, COVID-19 triggered communication and interaction among insurance companies and other stakeholders enabling information exchange and resource sharing that supported the development of the strategies in the three cities studied.

> *"I believe that when there is a common purpose and everyone is aware that it is a win-win, we agreed on the fundamentals, which was to think about people, in our city, it is the common purpose, that sense of belonging and that trust, is what allowed us to move forward" (Health Insurance representative, National Level, SH-D -006)*

Soft mechanism: growing confidence and trust in inter-organizational collaboration

Improved coordination between the different actors was facilitated by growing confidence and trust achieved through joint work as each organization realized that trading "blame" for collaboration would aid the achievement of their mutual objectives.

> *"I think that the key was, in the first place, understanding that it was a problem that affected everyone and that we all lost if we continued to blame ourselves. That was first and understanding that it was better to work together than to remain disjointed, blaming each other. Understand that protecting the community was most important. Save the lives of people who could be affected by the pandemic and that later when the pandemic passed, we could discuss the differences. That was a pact that we made between all of us, let's put politics and the discussions about money aside, and we are*

*going to concentrate on seeing how we all adjust and comply. And so, we began to work" ( Policymaker, Cartagena, SH-D-012)*

Soft mechanism: sense of shared responsibility for delivering coordinated surveillance

The agreements also created a fertile ground to plan and deliver coordinated surveillance activities. These initiatives facilitated the understanding of the COVID-19 response as a shared responsibility that exceeded the duty of each organization, allowing a collective approach to delivering services, rather than segmenting care by the patient´s insurance affiliation.

"*For the first time, we were able to coordinate different actors. This is the first time, that insurers and a regulatory body such as the secretariat of health, worked together to provide a response to a public health issue. This has been a coordinated response that has been based on the territory" (Secretariat of Health representative, Bogotá, SH-B-002)*

**Discussion**

This study identified structural and motivational barriers to inter-organizational coordination in the Colombian health system historically and indicated how COVID-19 triggered soft and hard mechanisms for working around these pre-existing barriers to support COVID-19 surveillance during the first year of the pandemic. Two propositions concerning critical factors that influence the coordination between institutions were introduced. The first proposition, concerning structural impediments to coordination, was supported by the qualitative data. Pre-existing structural barriers to coordination with the Colombian health system reported by the interviewees included: the fragmentation and segmentation of the health system, the major differences among multiple participant organizations, competition for market share among insurance companies, poorly defined roles, unfulfilled contractual agreements, and fragmented information systems.

The second proposition, concerning the importance of motivational or “soft” factors for supporting coordination, was found to be necessary but insufficient for enabling shared institutional surveillance of COVID-19. The magnitude of the pandemic, which threatened to overwhelm the capacity of Colombia´s public health surveillance system, became an incentive for cooperation. The growing appreciation of joint work was born from recognition that each organization was facing great challenges to fulfil their roles individually and that only by joining efforts it was possible to provide a response to the emergency. While the severity of the pandemic created “soft” pressure for increased coordination, a “hard” coordination mechanism – the formal institutional agreements – was needed to facilitate concrete practices of coordination.

This leads to a new proposition concerning coordination arrangements among institutions: *improving coordination between institutions relies on interplay among hard and soft coordination mechanisms*. As a hard mechanism of coordination, the formal agreements influenced practices of coordination in several ways. First, the agreements provided authority to negotiate new local arrangements among institutions. Negotiation of the local agreements was supported by a state of emergency declaration by national government (e.g República de Colombia, 2020b) which allowed for flexibility over organizational roles and functions, allowing local authorities to adapt and identify local solutions, and mobilized financial resources. With new-found legitimacy bestowed by the national declaration, local authorities were able to participate as mediators to legalize the agreements between insurance companies and health providers.

Second, the agreements formalized specific products of coordination. Organizations were able to share information about positive cases that needed tracing or information about patients that were at higher risk, and financial and human resources so they could implement the testing and tracing of cases beyond the insurance affiliation of the patient. The inter-organizational agreements, involving health secretaries, insurance companies and health providers in the three Colombian cities we studied, supported diagnostic testing in people´s homes, the rapid sharing of surveillance data, and community outreach work, all of which crossed traditional organizational boundaries within the health system.

Third, inter-organizational trust was improved as a by-product of the processes of collaboration instituted. Through the practice of forming and working in accordance with the formal agreements, insurance companies were able to work as allies instead of seeing each other merely as competitors. The initial pressure for, and subsequent process of collaboration, helped to address distrusting relationships among actors that had thwarted cooperative relationships historically within the Colombian health system.

**Contributions to the research literature**

This study contributes to two areas of research: structural influences on health system coordination and mechanisms for facilitating inter-organizational coordination. First, the international literature has identified structural barriers to inter-organizational coordination, including service fragmentation and competitive incentives associated with market-based public health systems like Colombia (Vargas et al. 2016). Absence of clear coordination mechanisms has been found to result in delayed response activities like testing and contact tracing in the context of the COVID-19 emergency (Tromberg et al., 2020). Our study contributes to this literature in highlighting how a wider range of structural barriers associated with market-based healthcare systems (poorly defined roles and relationships concerning coordination, unfulfilled contractual agreements, and fragmented information systems) affected early responses to COVID-19 in Colombia. Moreover, the findings indicate how structural barriers to coordination have knock-on effects on motivational barriers to coordination like distrusting relationships that stem from market-based behaviour. The findings contribute to this literature by indicating the need to address a wider range of structural barriers to coordination to influence, in turn, health system actors´ motivation to engage in coordination arrangements.

Second, this study contributes to debate on encouraging inter-organizational cooperation which has taken on increased importance in the context of COVID-19. It is recognised that soft incentives are needed to promote cooperation among actors and organizations such as institutional support and motivating professionals´ participation, especially in market-based systems like Colombia (Vargas et al. 2020). Our study similarly supports the need for soft mechanisms of coordination to address mutual distrust that may have developed as a by-product of systemic barriers. However, our findings suggest that soft mechanisms will have little impact on inter-organizational trust in the absence of harder mechanisms for promoting coordination. In the case of COVID-19, formal inter-organizational agreements covering shared roles and responsibilities, data management, and financial resources, were instrumental in facilitating joined up approaches to the surveillance of COVID-19. Together with the "permission" for innovation represented by COVID-19 (Swaithes et al. 2020), the process of negotiating formal inter-organizational agreements between organizations appeared to improve the quality of inter-personal relationships locally. While recognising the importance of inter-personal relationships for supporting coordination (Dickinson, 2010), the study deviates from previous literature on health system coordination that emphasizes "soft"

mechanisms exercised at the interpersonal level (e.g. political skill, trust-building exercises, stakeholder involvement) to improve coordination between organizations. Instead, the findings indicate the need to develop "hard" incentives to support collaboration as these were found to influence "soft" outcomes too (i.e. formal agreements or contractual mechanisms for coordination that formalize inter-organizational roles, resources, and relationships). The emphasis on "hard" mechanisms to support coordination complements previous work on major system change that includes a role for designated or top-down coordination in aligning muliple stakeholder interests (Turner et al. 2016a) and work in economics that places emphasis on formal and implicit contracts in shaping inter-organizational behaviour (Williamson, 1981; Williamson, 1991).

**Policy and practice implications**

The findings indicated, in this case, that "hard" intervention mechanisms supported improvement in the coordination relationships among institutions. While Government actions can be considered both as barriers and enablers to coordination depending on the circumstances (Casey, 2008), in the three cities we studied, interviewees recognized that formalization of relationships, achieved through "top-down" coordination by State actors at national and local level, were facilitating factors for improving coordination. Hard mechanisms such as formal agreements or contracts and regulatory change originated at the national level, providing the formal authority needed to improve collaboration among a variety of health system stakeholders both nationally and locally. The regulatory changes introduced by the national and local authorities enabled the development of collaborations initiatives among stakeholders both from the public and private sector. In market-based health systems in particular, policymakers need to consider the need for "top-down" intervention, expressed through contracting or regulatory mechanisms, where increased collaboration among institutions is required in response to immediate crises or longer-term system-wide challenges. While environmental disturbances like pandemics may trigger a disposure toward collaboration to address a crisis, formal intervention may well be needed to accomplish changes in relationships among organizations by aligning the multiple stakeholders involved.

The study showed that, to plan new strategies for surveillance based on coordinated work, understanding the influence of, and interplay between, soft and hard mechanisms on inter-organizational coordination in specific contexts is critical. Such understanding should be informed by qualitative studies which inquire into the sociocultural aspects that shape public health measures to mitigate pandemics (Bavel et al. (2020), Teti et al. (2020) and Vindrola-Padros et al. (2020). This study showed that the planning and implementation of surveillance strategies are deeply intertwined with local perceptions about what inter-organizational coordinated work means for different actors and the history of organizational relationships within local health systems.  In the case of the Colombian health system, we found that formalizing the Agreements facilitated sharing information, legal procedures, mobilizing financial resources. However, these processes also required acknowledgement of a new form of leadership locally (held by the health secretariats), and willingness to engage in inter-organizational relationships concerning COVID-19 surveillance, which were fueled by initial pressure to act, while trust then developed through the process of collaborative work. Before introducing major changes in the structure of health systems, public health planners should understand how mechanisms of change work at the local level to include incentives that can be properly adapted to the specific contexts of each city or region within the same country.

**Limitations**

We were unable to conduct face-to-face interviews given the quarantine restrictions taking place in Colombia during the study, although online interviews had a good participation rate (71%) and acquired a range of actors´ perspectives. Triangulating the actors´ reported experiences with observations of everyday interactions relevant for coordinated work (e.g. planning meetings) would be a valuable component of follow-up research that assesses barriers and facilitators to the sustaining of coordination arrangements post-COVID. Finally, the three cities have different local contexts, both socio-economic characteristics and health system arrangements, limiting cross-case comparability and generalizability of the findings to other settings. Moreover, while a range of factors influencing the implementation of coordination arrangements between organizations have been identified, it is not possible to isolate the relative role of softer and harder mechanisms (e.g. the effect of an institutional agreement relative to increased purposeful communications stimulated by the threat to health posed by COVID-19). The results obtained could inform questionnaire items used in further cross-sectional survey research.

**Data availability**

The data underlying this article are available in the article and in its online supplementary material.

Figure 1. Household social security affiliation diagram

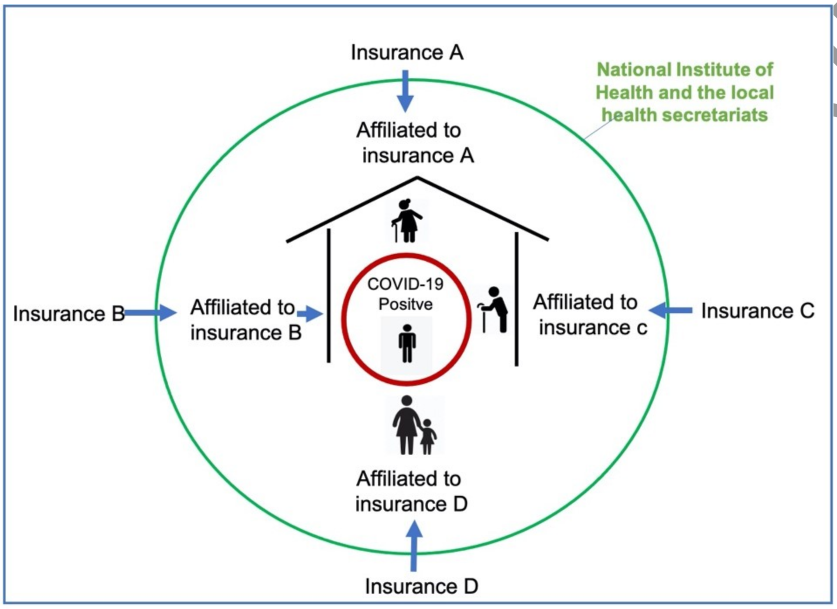

**Table 1. Characteristics of the Colombian health system of its response to COVID-19**

| |
|---|
| **Structure** |
| • Mixed system in which both the public and private sector participate in the insurance, management, delivery, and funding of health care services (Guerrero et al., 2011).<br>• Segmentation and fragmentation coexist in the Colombian healthcare system (Jaime, 2016, Hernández, 2002).<br>• Market oriented (Giovanella et al., 2020)<br>• Structured pluralism health model in which health services are highly privatised (Homedes and Ugalde, 2005) |
| **Coverage** |
| In Colombia, affiliation to the General Health Security System - SGSSS is composed by two insurance schemes. The contributory which covers formally employed and independent workers and the subsidised which covers individuals classified as poor according to a proxy means test (SISBEN (Escobar et al., 2009). |
| • Universal Health Coverage of 96% in 2016 (Ministerio de Salud y de la Protección Social, 2012). |

| **Financing sources** |
|---|
| • Colombia has a social health insurance model, funded through general taxation and payroll deductible contributions. |
| **COVID-19 preparedness** |
| • Declaration of the State of Economic, Social and Ecological Emergency in the country (República de Colombia, 2020c)<br>• National Contingency Plan (Ministerio de Salud y Protección Social, 2020)<br>• Decree 538 to strengthen the health services provided in Colombia for the management of COVID-19 (República de Colombia, 2020b) |

Table 2. Characteristics of interviewees

| Organisation | Structure of the health system | Type of organisation | N |
|---|---|---|---|
| National Government | National level - planning | Public | 9 |
| **CARTAGENA** | | | |
| Local Government | Local level - planning | Public | 9 |
| Health insurances, prepaid companies, | Insurance | Public and private | 6 |
| Hospitals | Service delivery | Private | 5 |
| Hospitals | | Public | 2 |
| Laboratories | | Public and private | 2 |
| **CALI** | | | |
| Local Government | Local level - planning | Public | 5 |
| Insurance companies, prepaid companies | Insurance | Public and private | 2 |
| Hospitals | Service delivery | Private | 10 |
| Hospitals | | Public | 2 |
| Laboratories | | Public and private | 2 |
| **BOGOTA** | | | |

| | | | |
|---|---|---|---|
| Local Government | Local level - planning | Public | 8 |
| Insurance companies, prepaid companies | Insurance | Public and private | 5 |
| Hospitals | | Private | 8 |
| Hospitals | Service delivery | Public | 4 |
| Laboratories | | Public and private | 2 |
| | | **Total** | 81 |

**Table 3**. List of codes, sub codes and emerging categories.

| **Key Code** | **Sub-Codes** | **Emergin codes relevant to understand inter-organizational coordination** |
|---|---|---|
| Organization Response | Decision making processes | - - - |
| | Organization priorities | |
| | Enablers (Financial  legal, social relations, information access and use) | |
| | Pre-existing conditions | |
| | Barriers (Financial  legal, social relations, information access and use) | |
| | Challenges | |
| | Infrastructure / installed capacity | |
| | Human resources | |
| | Forms of innovation  (processes, productios, relationships, services) | |
| | Indirect impact in other services | |
| Coordination and cooperation | National and local goverment relationship | |
| | Inter organization involvement | Legal framworks (Convenio tripartita) |
| | | Responsability distribution |
| | Public and private relationships | Resource allocation |
| | Pre-existing conditions | - - - |
| | Leadership | |
| | Intersectoral involvement | |
| Mayor System Change theory | Feedback loops | |
| | Health system stakehodler engagement | Communication |

| | | |
|---|---|---|
| | Implementing change | - - - |
| | Learning from history | |
| Levers | Hard Levers | |
| | Soft Levers | |
| Health System Charactersitics | - - - | Fragmentation |
| | | Segmentation |
| | | Trust |
| | | Surveillance system operation |
| | | Service planning and implementation |
| | | Information Systems |
| Corruption | - - - | - - - |
| Expertise Narratives | - - - | |
| Social determinants of health | - - - | |
| Technology use and appropiation | - - - | |
| Evidence production and use | - - - | |

**Table 4.** Public health survillance responsibilities in the Colombian healthcare system

| Activities | Responsible |
|---|---|
| Public health surveillance | National health Institute (INS), Departments and municipalities (local health secretaries) |
| Sample testing | Public and private health providers (private clinical laboratories and health secretaries) |
| Payers (who pay for the service) | Health insurance companies (EPS), prepaid insurance companies, for patients with no payment capacity: public health services |
| Enforcement of health regulations and insurance contracts | Ministry of Health |

**Topic guide for stakeholder interviews**

Major system change involves coordinated action by health care planners and providers to improve services across a whole health system (e.g. a metropolitan area such as Bogota). This case study focuses on the response of health planners and providers to COVID-19 within different local health sytems across Colombia. The purpose of these interviews is to establish stakeholders' priorities for health systems research on, and feedback approaches to support, the response to COVID-19. The bullet points are prompts, where needed *

1. PLease, tell us about your **profesional background**, role and functions in the organisation.

2. What has been the **organisation's experience** with the pandemic during the past months?
    - Key challenges
    - Changes over time (national phase, LATAM & local prevalence, evidence availability)
    - Preparedness to respond to the pandemic (i.e preconditions of organisation)

3. What mechanisms have you put in place to identify, interpret and apply emerging evidence on COVID-19? What is key, what is lacking?
    - Source (e.g. WHO, national, regional, internal, other health systems)
    - Type (audit data, research articles, clinical guidance, media reporting, protocols, legal rules).

4. Do you have adequate **resources** for responding to COVID-19?
    - Staff
    - Physical space
    - Equipment
    - Funding
    - Processes to support operations (including procurement / supply chain)

5. What **plans** do you have for changing capacity, if necessary?
    - Humanitarian plan actions
    - Provisional care units
    - Changing roles / responsibilities over time

6. **Which other organisations are you working with** in planning and delivering care (pre-existing and COVID-19 specific)?
    - Planning
    - Delivery
    - **Public-private relationships** (e.g. bed capacity/seconding staff/technical knowledge/ sharing equipment, e.g. Personal Protective Equipment (PPE), ventilators, oxygen)

7. Which **relationships** are working well, and where could coordination be improved?
    - Internal
    - External (including municipalites/local government and national government agencies)
    - Public-private relationships

8. What **changes** has your department or organisation made to address COVID-19?

- Indirect impact in other Health services.

9. ¿ Do you have in mind an **example of practices** that have been giving solutions to address COVID 19?

10. What are the **organization's priorities** for improving the response to COVID-19?

11. What are the **wider health system priorities** across Bogotá (or Cali/Cartagena) for responding to COVID-19?

12. Following the pandemic, how do you consider that the **future** leadership, funding, planning and delivery of health and care services will be affected? Which adaptations to the health system will be useful for the future?

13. What **feedback** from this study would be most useful to you? How? Do you suggest any example?
    - Types of information / Communication method / overcoming implementation issues, e.g. technical capacity in public health
    - Your individual point of view
