# Supplementary material for: Implementing COVID-19 surveillance through inter-organizational coordination: a qualitative study of three cities in Colombia
Source: Health Policy Plan. 2021 Dec 7;37(2):232–42. doi: 10.1093/heapol/czab145 (PMC8689710; doi:10.1093/heapol/czab145)
Supplement: czab145_Supp [file czab145_supp.zip › Topic guide .docx]

**Topic guide for stakeholder interviews**

Major system change involves coordinated action by health care planners and providers to improve services across a whole health system (e.g. a metropolitan area such as Bogota). This case study focuses on the response of health planners and providers to COVID-19 within different local health sytems across Colombia. The purpose of these interviews is to establish stakeholders' priorities for health systems research on, and feedback approaches to support, the response to COVID-19. The bullet points are prompts, where needed *

1. PLease, tell us about your **profesional background**, role and functions in the organisation.
2. What has been the **organisation's experience** with the pandemic during the past months?
   - Key challenges
   - Changes over time (national phase, LATAM & local prevalence, evidence availability)
   - Preparedness to respond to the pandemic (i.e preconditions of organisation)
3. What mechanisms have you put in place to identify, interpret and apply emerging evidence on COVID-19? What is key, what is lacking?
   - Source (e.g. WHO, national, regional, internal, other health systems)
   - Type (audit data, research articles, clinical guidance, media reporting, protocols, legal rules).
4. Do you have adequate **resources** for responding to COVID-19?
   - Staff
   - Physical space
   - Equipment
   - Funding
   - Processes to support operations (including procurement / supply chain)

1. What **plans** do you have for changing capacity, if necessary?
   - Humanitarian plan actions
   - Provisional care units
   - Changing roles / responsibilities over time
2. **Which other organisations are you working with** in planning and delivering care (pre-existing and COVID-19 specific)?
   - Planning
   - Delivery
   - **Public-private relationships** (e.g. bed capacity/seconding staff/technical knowledge/ sharing equipment, e.g. Personal Protective Equipment (PPE), ventilators, oxygen)
3. Which **relationships** are working well, and where could coordination be improved?
   - Internal
   - External (including municipalites/local government and national government agencies)
   - Public-private relationships
4. What **changes** has your department or organisation made to address COVID-19?
   - Indirect impact in other Health services.
5. ¿ Do you have in mind an **example of practices** that have been giving solutions to address COVID 19?
6. What are the **organization's priorities** for improving the response to COVID-19?
7. What are the **wider health system priorities** across Bogotá (or Cali/Cartagena) for responding to COVID-19?
8. Following the pandemic, how do you consider that the **future** leadership, funding, planning and delivery of health and care services will be affected?  Which adaptations to the health system will be useful for the future?
9. What **feedback** from this study would be most useful to you? How? Do you suggest any example?
   - Types of information / Communication method / overcoming implementation issues, e.g. technical capacity in public health
   - Your individual point of view
